# Adolescent alcohol and cannabis use and early adulthood educational attainment in the 1986 Northern Finland birth cohort study

**DOI:** 10.1186/s12889-024-17693-w

**Published:** 2024-01-22

**Authors:** Jonna Levola, Anni-Emilia Alakokkare, Alexander Denissoff, Antti Mustonen, Jouko Miettunen, Solja Niemelä

**Affiliations:** 1 Department of Psychiatry, University of Helsinki, F1-00014 Helsinki, P.O. Box 63, Finland; 2 Psychiatry, Hospital District of Helsinki and Uusimaa, Helsinki, Finland; 3 Research Unit of Population Health, University of Oulu, Oulu, Finland; 4 Addiction Psychiatry Unit, Department of Psychiatry, Turku University Hospital, The wellbeing services county of Southwest Finland, Turku, Finland; 5 Department of Psychiatry, University of Turku, Turku, Finland; 6 Faculty of Medicine and Health Technology, Tampere University, Tampere, Finland; 7 Department of Psychiatry, Seinäjoki Central Hospital, Seinäjoki, Finland; 8 Medical Research Center Oulu, Oulu University Hospital and University of Oulu, Oulu, Finland

**Keywords:** Alcohol, Cannabis, Educational attainment, Intoxication, Adolescence, Birth-cohort study

## Abstract

**Background:**

Heavy alcohol and cannabis use during adolescence have been previously described as risk factors not only for morbidity in adulthood, but also social problems including adversities in educational attainment. Attempts to consider overlapping risk factors and confounders for these associations are needed.

**Methods:**

Using weighted multivariable models, we examined prospective associations between age at first drink (AFD), age at first intoxication (AFI), frequency of alcohol intoxication, as well as self-reported alcohol tolerance (i.e., number of drinks needed for the subjective experience of intoxication), and lifetime cannabis use at age 15/16 years with subsequent educational attainment obtained from comprehensive registers until age 33 in the Northern Finland Birth Cohort 1986 (6,564 individuals, 49.1% male). Confounding variables including sex, family structure (intact vs. non-intact), maternal and paternal education level, behavioural/emotional problems in school at age 7/8 years, having a history of illicit substance use in adolescence, having any psychiatric diagnosis before age 16, and parental psychiatric diagnoses, were adjusted for.

**Results:**

In this large birth cohort study with a 17-year follow-up, younger age at first intoxication, higher frequency of alcohol intoxication, and high self-reported alcohol tolerance at age 15/16 years were associated with poorer educational outcomes by the age of 33 years. These associations were evident regardless of potential confounders, including parental education and childhood behavioural/emotional problems. The association between adolescent cannabis use and educational attainment in adulthood was no longer statistically significant after adjusting for confounders including frequency of alcohol intoxication at age 15/16.

**Conclusions:**

Assessments of age of first alcohol intoxication, high self-reported alcohol tolerance and frequency of intoxication during adolescence should be included when implementing screening strategies aimed at identifying adolescents at risk for subsequent social problems.

**Supplementary Information:**

The online version contains supplementary material available at 10.1186/s12889-024-17693-w.

## Background

Alcohol and cannabis are the most commonly used substances in adolescence together with tobacco [1]. Adolescent substance use is a risk factor for long-term adverse outcomes, such as alcohol and/or substance use disorders [2, 3], self-harm [4, 5], premature mortality [6, 7], and social problems including unemployment [8–11]. Previous longitudinal studies have also indicated that adolescent substance use has an impact on educational outcomes both in adolescence, but also later in life [8, 12, 13]. The sequalae of adolescent substance use are a major concern and further research specifically among long-term prospective cohort studies has been called for [14].

When studying the sequelae of adolescent alcohol use, age at first drink (AFD) and age at first intoxication (AFI) have been a common focus of interest. Both AFD and AFI have been associated with adversities later in life (e.g., 2, 4, 6), however, results regarding educational attainment remain somewhat mixed with some studies reporting no association after confounding is adjusted for [15], and others reporting adverse associations in certain subgroups, such as working class males [16].

Binge drinking, most often defined as consuming a large amount of alcohol in a defined period of time, in adolescence has also been associated with various adverse outcomes in adulthood in previous literature, including psychiatric morbidity, convictions, lower social class and underachieving [17]. Previous studies examining the consequences of adolescent binge drinking have focused typically on number of drinks were consumed rather than on the individual’s experience. Self-reported tolerance refers to an individual’s subjective experience of the number of drinks needed to feel intoxicated. Studying subjective experience of intoxication could potentially capture young drinkers who can become intoxicated with fewer drinks than the typical definitions of binge drinking and are at risk both for acute and long-term harm [4, 7]. In previous literature, high inherent alcohol tolerance in adolescence has been found to be associated with subsequent AUD in and all-cause mortality in early adulthood, as well as self-harm and suicide [3, 4, 7, 18]. To the authors knowledge, no birth cohort studies have previously examined the association of inherent alcohol tolerance in adolescence with subsequent educational attainment.

Cannabis intoxication is associated with perturbations of several cognitive domains [19]. Even though cognitive decline in adolescence has not been shown to persist after protracted abstinence [20], it is plausible that weekly or daily use might perturb academic performance. This is supported by the evidence from some longitudinal and twin studies, which have found that cannabis use is associated with non-completion of secondary school compared to non-using controls [12, 21]. Whether this association of cannabis use in adolescence and poorer educational attainment is causal i.e., due to cannabis use, or reflects overlapping risk factors for both cannabis use and disengagement form school and studies, is somewhat unclear.

The current study aimed to investigate associations of adolescent alcohol and cannabis use with subsequent educational attainment. We examined the prospective association between (1) AFD, (2) AFI, (3) frequency of alcohol intoxication, as well as (4) self-reported alcohol tolerance (i.e., number of drinks needed for the subjective experience of intoxication) at age 15/16 years, and (5) lifetime cannabis use by age 15/16 and with subsequent educational attainment until age 33 in the Northern Finland Birth Cohort Study 1986 (NFBC1986). To the authors’ knowledge, no previous studies have examined associations of all of these various alcohol use variables, including self-reported alcohol tolerance in adolescence, with subsequent educational attainment, nor the possible confounding of associations of adolescent alcohol and cannabis use.

## Methods

The NFBC 1986 is an ongoing multidisciplinary birth cohort study comprising 99% of all live-born children (*n* = 9432) with an expected date of birth between July 1, 1985, and June 30, 1986, from the two northernmost provinces in Finland [22, 23]. Parents and offspring have been followed-up in regular intervals including clinical studies at child ages 7/8 years and 15/16 years old. This study concerns the data collected in 2001–2002 when study members were aged 15/16 years. The data collection for the adolescent follow-up entailed participants and their parents with known addresses (*n* = 9215). Participants who themselves and whose parents had given informed consent for the follow-up (*n* = 7760; 49.8% male) were included in the current study (flowchart of study, please see supplement 1).

The study was approved by the Northern Ostrobothnia Hospital District Ethical Committee 108/2017 (15.1.2018). The authors assert having followed STROBE guidelines for cohort studies [24].

### Measures

#### Outcome: educational attainment

Educational level was obtained from the national education register via linking the participants unique national identification numbers up to when the participants were 33 years old. Education was classified according to completion of Finnish mandatory primary school corresponding to 9 years of studies, obtaining a secondary degree (high-school or vocational studies) corresponding to approx. 12 years of studies, and higher education i.e., obtaining a college or university degree corresponding to over 12 years of studies. For the final analysis, due to their small number, those who had not completed primary school or for whom data regarding education was missing were pooled with those participants with primary school only.

#### Alcohol and cannabis use

The data on alcohol and drug use was collected using questionnaires that the participants received during the field study in adolescence. The participants were asked at what age did they first drink the following alcoholic beverages: beer, wine, spirits. The response options were never; at age 11 or under; at age 12; 13; 14; 15 or 16. Based on these answers, a variable designating the age when any alcoholic beverage was consumed for the first time, was formed. The participants were also asked at what age they had first been intoxicated (same response options). Alcohol intoxication was determined in this study according to self-report i.e., no set cut-off for the amount of alcohol was used.

For both of these variables, three groups were formed; those who had had their first drink or had first been intoxicated at age ≤ 14 or > 14, and the reference group of participants who had never consumed alcohol or had never been intoxicated. Age 14 is often considered a boundary between early and middle adolescence [25].

The participants were asked how many times in the previous 30 days they had been intoxicated. Frequency of intoxication was classified into three groups: infrequent intoxication (1–2 times in the previous 30 days) and frequent intoxication (≥ 3 times in the previous 30 days). Participants who had not consumed alcohol or had not been intoxicated in the past 30 days were considered the reference group.

In order to determine the level of alcohol tolerance, the participants were asked how many drinks they needed to feel intoxicated. A visual depiction of a standard Finnish drink (12 g of pure alcohol) was given. High alcohol tolerance was defined to correspond to the top 5 percentile and defined as ≥ 9 drinks for males and ≥ 7 drinks for females. Individuals with lower reported tolerance formed a second group. The third group included participants who had never consumed alcohol or had never been intoxicated and they were considered the reference group.

Data on frequency of lifetime cannabis use by 15/16 years was collected. The participants were asked whether they had ever used marihuana or hashish with options: never, once, 2–4 times, 5 times or more, or I use regularly. The participants were categorized into three groups: those adolescents who reported no cannabis use, those with one to four instances of use, and those with five or more instances of cannabis use.

### Confounding variables

#### Parental education level and family structure

There is strong evidence of intergenerational persistence of educational attainment [26], as well as the association with family structure [27]. These were controlled for in this study. Parental education was categorized into ≥ 12 years corresponding to vocational or university studies and < 12 years corresponding to primary school without a secondary degree. Information on family structure was gathered by combining information collected from parents at birth and when the cohort member was an adolescent. Family structure was classified as (a) intact (both parents living with the participant all the time) and (b) non-intact (one parent or other families).

#### Psychiatric diagnoses

Of all health problems in adolescence, mental health disorders are most strongly associated with poor educational outcomes [28]. Diagnoses of the adolescents’ psychiatric disorders (F00-99), excluding substance use disorders by age 15/16 according to the International Classification of Disease, 10th revision (ICD-10) [29] were obtained from four national registers: outpatient registers for primary and specialized care, the Care Register for Health Care, disability pensions of the Finnish Centre for Pensions and the medication reimbursement register of the Social Insurance Institution of Finland. The Care Register contains information on patients discharged from inpatient care, and since 1998 also on specialized outpatient care. The Register of Primary Health Care Visits includes all outpatient primary health care delivered in Finland.

There is some evidence that some parental mental health problems, specifically maternal anxiety, is associated with poor educational attainment in the offspring [30]. Thus, parental psychiatric disorders were also included as confounders in this study. Parental psychiatric diagnoses were obtained from the same registers, except for the medication reimbursement register, up to the year 2001 when the participants were 15/16 years, and the clinical study was conducted.

#### Behavioural and emotional problems in school at age 7/8

Behavioural and/or emotional problems, both internalizing and externalizing, at ages 6 through 8 have been found to significantly and strongly diminish the probability of receiving a high school degree [31]. Behavioural/emotional problems in the classroom, such as difficulties concentrating, restlessness and anxiety, were evaluated by the participants’ teachers when the participants were 7/8 years old. The Rutter scale [32] was used to differentiate between individuals with clinically relevant behavioural/emotional problems in the school environment using a cut-off recommended by Rutter et al. of nine or more points.

#### Smoking and illicit substance use

Both smoking and illicit substance use in adolescence are associated with adverse educational outcomes and unemployment later in life [10, 15]. Information on smoking was ascertained in postal questionnaires and data on illicit substance use were collected via questionnaire that the participants completed during the field study. Information on tobacco smoking was studied and the participants were categorized into ‘daily smoking (no/yes)’. Data on illicit substance use were collected with several questions (no/yes) concerning, for example, cannabis use, prescription drug use, use of inhalants and other illicit drugs. These were combined as ‘Illicit substance use (no/yes)’.

### Statistical analyses

Cross-tabulation and Chi-square-tests were used for studying the associations of background information and alcohol and cannabis use variables with educational attainment. Multivariable logistic regression models were used to analyse differences between the groups categorized according to educational attainment according to AFD, AFI, frequency of intoxication, alcohol tolerance and cannabis use. The confounding background variables based on previous literature were sex, family structure (intact vs. non-intact), maternal and paternal education (over or under 12 years), behavioural/emotional problems at age 7/8, having a history of illicit substance use in adolescence, having any psychiatric diagnosis before age 16, and parental psychiatric diagnoses. These confounding variables were also associated with educational attainment and at least one of the alcohol or cannabis use variables in this study (*p* < 0.1).

Multivariable logistic regression models were created to evaluate the predictive significance the alcohol (model 1) and cannabis use (model 2) variables on the outcome variable i.e., educational attainment, after adjusting for confounders. First in block 1, a set of confounding background variables related to the participants themselves was added to the models (sex, behavioural/emotional problems at age 7/8 (Ruttery ≥ 9), any psychiatric diagnosis by 2018 and lifetime cannabis or other illicit substance use in model 1 and frequency of alcohol intoxication at age 15/16 in model 2). Second in block 2, family-related confounding variables were added (family type, maternal and paternal education and parental psychiatric disorder). Lastly in block 3, alcohol (model 1) or cannabis related (model 2) variables were added. Because all of the alcohol and cannabis use related variables were statistically significantly associated with each other (*p* < 0.0001), separate fully adjusted models were created for the dependent variables: age at first drink (block 3a), first alcohol intoxication (block 3b), frequency of intoxication (block 3c) and self-reported alcohol tolerance (block 3d). ORs with 95% CIs were calculated with secondary school as the reference category, to which primary school only and college/university education were compared.

An analysis of attrition for this sample of the NFBC1986 has been previously described [33]. Males were less likely to participate in the adolescent follow-up than females (67% v. 74%; χ2 test, *p* < 0.001). Also, adolescents with a maternal (65% v. 72%, *p* < 0.001) or paternal (71% v. 81%, *p* < 0.001) history of psychiatric disorders were less likely to participate than others. To account for this attrition due to non-participation, we weighted our multivariable analyses by sex, parental psychiatric disorder, and urbanicity by using inverse probability weighting [34].

All results were considered statistically significant at *p* < 0.05. Statistical analyses were performed with SPSS v. 28.0.1.

## Results

Of the 7760 adolescents (49.8% males), 0.4% had not completed primary education or data on education was missing (Table 1). Primary school only was completed by 5.7% (8.1% males, 3.3% females). In all, 48.0% (55.0% males, 49.0% females) had obtained a secondary education, and 46.0% (36.4% males, 55.4% females) a college or university degree. Sex, family type, behavioural/emotional problems at age 7/8 years, maternal and paternal education, parental psychiatric diagnoses, AFD, AFI, frequency of intoxication as well as self-reported alcohol tolerance, and daily smoking were all statistically significantly associated with educational level. Univariate associations with cannabis or other illicit drug use and educational level could not be analysed due to small subgroup sizes.

**Table 1:** Association of background information, alcohol and substance use in adolescence and subsequent educational attainment

		Educational attainment			
	All n (% of all)	No primary education or data missing n (%)	Primary education n (%)	Secondary education/ degree n (%)	College/ university education/ degree n (%)
**All**	**7760 (100.0)**	34 (0.4)	439 (5.7)	3721 (48.0)	3566 (46.0)
**Deceased or immigrated (% of all)**	391 (5.0)	7 (20.6)	57 (13.0)	156 (4.2)	171 (4.8)
Sex***	**7760 (100.0)**				
Male	3865 (49.8)	19 (0.5)	312 (8.1)	2126 (55.0)	1408 (36.4)
Female	3895 (50.2)	15 (0.4)	127 (3.3)	1595 (40.9)	2158 (55.4)
Family type***	**6591 (84.9)**				
Two parents	5091 (77.2)	23 (0.5)	213 (4.2)	2256 (44.3)	2599 (51.1)
One parent or other	1500 (22.8)	3 (0.2)	116 (7.7)	811 (54.1)	570 (38.0)
Maternal education***	**6559 (84.5)**				
≥ 12 years	2126 (32.4)	5 (0.2)	58 (2.7)	700 (32.9)	1363 (64.1)
< 12 years	4433 (67.6)	22 (0.5)	270 (6.1)	2355 (53.1)	1786 (40.3)
Paternal education***	**6262 (80.7)**				
≥ 12 years	1184 (18.9)	4 (0.3)	35 (3.0)	346 (29.2)	799 (67.5)
< 12 years	5078 (81.1)	22 (0.4)	268 (5.3)	2554 (50.3)	2234 (44.0)
Parental psychiatric disorder***	**7760 (100.0)**				
No	4824 (62.2)	19 (0.4)	211 (4.4)	2219 (46.0)	2375 (49.2)
Yes	2936 (37.8)	15 (5.1)	228 (7.8)	1502 (51.2)	1191 (40.1)
Behavioural/emotional problems at age 7/8***	**7140 (92.1)**				
No (Rutter < 9)	6183 (86.6)	17 (0.3)	271 (4.4)	2833 (45.8)	3062 (49.5)
Yes (Rutter ≥ 9)	957 (13.4)	8 (0.8)	124 (13.0)	570 (59.6)	255 (26.6)
Age at first drink***	**6631 (85.5)**				
No alcohol use until age 15/16	1423 (21.5)	11 (0.7)	51 (3.6)	608 (42.7)	753 (52.9)
> 14 years	538 (8.1)	3 (0.6)	22 (4.1)	237 (44.1)	276 (51.3)
≤ 14 years	4670 (70.4)	4 (0.1)	247 (5.3)	2252 (48.2)	2167 (46.4)
Age at first intoxication***	**6565 (84.6)**				
No alcohol use / intoxication until age 15/16	2165 (33.0)	13 (0.6)	76 (3.5)	909 (42.0)	1167 (53.9)
> 14 years	1085 (16.5)	2 (0.2)	45 (4.1)	502 (46.3)	536 (49.4)
≤ 14 years	3315 (50.5)	3 (0.1)	196 (5.9)	1654 (49.9)	1462 (44.1)
**Frequency of alcohol intoxication** ^**1**^ ***	**6462 (83.3)**				
0 times	3861 (59.7)	13 (0.3)	151 (3.9)	1662 (43.0)	2035 (52.7)
1–2 times	1967 (30.4)	2 (0.1)	99 (5.0)	954 (48.5)	912 (46.4)
≥ 3 times	634 (9.8)	2 (0.3)	63 (9.9)	384 (60.6)	185 (29.2)
**Alcohol tolerance** ^**2**^ ***	**6615 (85.2)**				
No alcohol use / intoxication until age 15/16	2119 (32.0)	13 (0.6)	75 (3.5)	890 (42.0)	1141 (53.8)
< 9 (males) / <7 (females)	3733 (56.4)	5 (0.1)	161 (4.3)	1717 (46.0)	1850 (49.6)
≥ 9 (males) / ≥7 (females)	763 (11.5)	0 (0.0)	83 (10.9)	482 (63.2)	198 (26.0)
Daily smoking at age 15/16***	**6955 (89.6)**				
No	6019 (86.5)	21 (0.3)	259 (4.3)	2615 (43.4)	3124 (51.9)
Yes	936 (13.5)	1 (0.1)	98 (10.5)	630 (67.3)	207 (22.1)
**Lifetime cannabis use** ^**3**^	**6586 (84.9)**				
No cannabis use until age 15/16	6209 (94.3)	18 (0.3)	278 (4.5)	2890 (46.5)	3023 (48.7)
1–4 times	311 (4.7)	0 (0.0)	29 (9.3)	154 (49.5)	128 (41.2)
≥ 5 times	66 (1.0)	0 (0.0)	12 (18.2)	37 (56.1)	17 (25.8)
**Lifetime other illicit substance use** ^**3**^	**6627 (85.4)**				
No other illicit substance use until age 15/16	5912 (89.2)	18 (0.3)	272 (4.6)	2702 (45.7)	2920 (49.4)
Yes	715 (10.8)	0 (0.0)	49 (6.9)	394 (55.1)	272 (38.0)
**Any psychiatric diagnosis by 2018**	**7760 (100.0)**				
No	5882 (75.8)	29 (0.5)	252 (4.3)	2735 (46.5)	2866 (48.7)
Yes	1878 (24.2)	5 (0.3)	187 (10.0)	986 (52.5)	700 (37.3)

^1^ Past 30 days ^2^ Self-reported number of standard Finnish drinks (12 g) required for inebriation ^3^ Unable to calculate due to zero cells

* *p* < 0.05 **p < 0.01 ***p < 0.001

In the multivariable models, females were less likely than males to complete only primary education compared to secondary education (OR 0.53; 95% CI 0.37–0.74; *p* < 0.001). Behavioural/emotional problems at age 7/8 years (OR 2.21; 95% CI 1.59–3.06; *p* < 0.001) and psychiatric diagnoses by age 15/16 years (OR 2.98; 1.78–4.98; *p* < 0.001) were associated with statistically significantly increased ORs for completing only primary education compared to secondary education. (Table 2; model 1, block 1)

**Table 2:** Association of adolescent alcohol and cannabis use with subsequent educational attainment, odds ratios (ORs) compared with secondary school degree

	MODEL 1 Alcohol					MODEL 2 Cannabis			
	Primary education or less / missing data		College / university education /degree			Primary education or less		College / university education /degree	
	OR (95% CI)	*p*	OR (95% CI)	*p*		OR (95% CI)	*p*	OR (95% CI)	*p*
**Block 1**					**Block 1**				
Female sex	0.53 (0.37–0.74)	***< 0.001***	2.08 (1.84–2.35)	***< 0.001***	Female sex	0.48 (0.34–0.69)	***< 0.001***	2.04 (1.81–2.31)	***< 0.001***
Behavioural/emotional problems at age 7/8 (Rutter ≥ 9)	2.21 (1.59–3.06)	***< 0.001***	0.49 (0.40–0.59)	***< 0.001***	Behavioural/emotional problems at age 7/8 (Rutter ≥ 9)	2.14 (1.53–2.99)	***< 0.001***	0.47 (0.38–0.58)	***< 0.001***
Any psychiatric diagnosis by 2018	2.98 (1.78–4.98)	***< 0.001***	0.52 (0.35–0.77)	***0.001***	Any psychiatric diagnosis by 2018	3.08 (1.83–5.19)	***< 0.001***	0.47 (0.32–0.69)	***< 0.001***
Lifetime cannabis or other illicit substance use	1.20 (0.79–1.81)	0.398	0.68 (0.56–0.82)	***< 0.001***	Frequency of alcohol intoxication past 30 days *(ref. none)*				
					1–2 times	1.21 (0.86–1.70)	0.271	0.79 (0.69–0.91)	***0.001***
					3 times	1.94 (1.28–2.94)	***0.002***	0.41 (0.32–0.51)	***< 0.001***
**Block 2**					**Block 2**				
Non-intact family type	1.17 (0.75–1.83)	0.494	0.73 (0.60–0.88)	***0.001***	Non-intact family type	1.13 (0.72–1.77)	0.590	0.75 (0.62–0.92)	***0.005***
Maternal education ≥ 12 years	1.09 (0.70–1.71)	0.693	2.23 (1.90–2.61)	***< 0.001***	Maternal education ≥ 12 years	1.15 (0.74–1.81)	0.530	2.17 (1.85–2.54)	***< 0.001***
Paternal education ≥ 12 years	0.84 (0.44–1.60)	0.593	2.16 (1.76–2.64)	***< 0.001***	Paternal education ≥ 12 years	0.86 (0.45–1.64)	0.645	2.17 (1.76–2.66)	***< 0.001***
Parental psychiatric disorder	1.47 (1.02–2.12)	***0.041***	0.90 (0.78–1.04)	0.167	Parental psychiatric disorder	1.41 (0.97–2.05)	0.073	0.88 (0.75–1.02)	0.085
**Block 3a**					**Block 3**				
Age at first drink (*ref. never)*					Lifetime cannabis use *(ref. never)*				
> 14 years	1.04 (0.46–2.33)	0.934	1.11 (0.84–1.47)	0.451	1–4 times	0.49 (0.17–1.41)	0.183	1.03 (0.71–1.50)	0.871
≤ 14 years	1.23 (0.75–2.01)	0.422	0.92 (0.78–1.09)	0.342	≥ 5 times	2.22 (0.61–8.09)	0.229	0.88 (0.36–2.17)	0.780
**Block 3b**									
Age at first intoxication *(ref. never)*									
> 14 years	1.07 (0.60–1.92)	0.821	0.85 (0.69–1.04)	0.116					
≤ 14 years	1.38 (0.88–2.14)	0.158	0.70 (0.60–0.82)	***< 0.001***					
**Block 3c**									
Frequency of alcohol intoxication past 30 days *(ref. none)*									
1–2 times	1.30 (0.86–1.98)	0.209	0.78 (0.67–0.92)	***0.002***					
≥ 3 times	2.00 (1.15–3.48)	***0.014***	0.42 (0.32–0.56)	***< 0.001***					
**Block 3d**									
Alcohol tolerance *(ref. no use/intoxications)*									
< 9 (males) / <7 (females)	1.19 (0.76–1.87)	0.450	0.85 (0.73–0.99)	***0.038***					
≥ 9 (males) / ≥7 (females)	2.10 (1.21–3.65)	***0.009***	0.40 (0.30–0.52)	***< 0.001***					

Female sex (OR 2.08; 95% CI 1.84–2.35; *p* < 0.001) and both maternal (OR 2.23; 95% CI 1.90–2.61; *p* < 0.001) and paternal (OR 2.16; 95% CI 1.76–2.64; *p* < 0.001) higher education were associated with statistically significantly increased ORs for college/university education compared to secondary education (Table 2; model 1, blocks 1and 2). Behavioural/emotional problems at age 7/8 years (OR 0.49; 95% CI 0.40–0.59; *p* < 0.001), psychiatric diagnoses by age 15/16 years (OR 0.52; 95% CI 0.35–0.77; *p* = 0.001), and non-intact family structure (OR 0.73; 95% CI 0.60–0.88; *p* = 0.001) were associated with statistically significantly decreased ORs for college/university education compared to secondary education (Table 2; model 1, blocks 1 and 2).

Frequent alcohol intoxication (more than three times in past 30 days) at age 15/16 years (OR 2.00; 95% CI 1.15–3.48; *p* = 0.014) and high self-reported alcohol tolerance (< 9 alcohol units for males, < 7 for females; OR 2.10; 95% CI 1.21–3.65; *p* = 0.009) were associated with increased ORs of completing primary education only compared to secondary education (Table 2; model 1, blocks 3c and 3d). AFI (OR 0.70; 95% CI 0.60–0.82; *p* < 0.001), alcohol intoxication frequency during the past month at age 15/16 (1–2 times OR 0.78; 95% CI 0.67–0.92; *p* = 0.002; 3 or more times OR 0.42; 95% CI 0.32–0.56; *p* < 0.001) and high self-reported alcohol tolerance (OR 0.40; 95% CI 0.30–0.52 *p* < 0.001) were associated with decreased ORs for college/university education compared to secondary education (Table 2; model 1, blocks 3b, 3c and 3d). Notably, also those reporting lower alcohol tolerance were less likely to complete college/university education compared to secondary education when compared to those with no alcohol use or never been intoxicated (OR 0.85; 95% CI 0.73–0.99; *p* = 0.038).

Cannabis use by age 15/16 years was not associated with of completing primary education only compared to secondary education. Lifetime use of cannabis or other illicit substance use (yes/no) was associated with lower odds of college/university education compared to secondary education (OR 0.68; 95% CI 0.56–0.82; *p* < 0.001) when sex, behavioural/emtional problems at age 7/8 years and psychiatric diagnoses by age 15/16 years were controlled for (Table 2: model 1, block 1). However, when family-related variables and frequency of alcohol intoxication at age 15/16 years were controlled for, cannabis use was no longer statistically significantly associated with lower odds of college/university education compared to secondary education (1–4 times OR 1.03; 95% CI 0.71–1.50; *p* = 0.871; 5 times or more OR 0.88; 95% CI 0.36–2.17; *p* = 0.780). (Table 2: mode 2, block 3).

## Discussion

In this large birth cohort study with a 17-year follow-up, younger age at first intoxication, higher frequency of alcohol intoxication, and high self-reported alcohol tolerance at age 15/16 years were associated with poorer educational outcomes by the age of 33 years. These adverse associations were evident regardless of a range of potential confounders, such as behavioural/emotional problems at age 7/8 years and parental education level. The association between adolescent lifetime cannabis use and educational attainment in adulthood was no longer statistically significant after adjusting for potential confounders including alcohol use.

Our finding that inherent alcohol tolerance in adolescence was associated with subsequent educational attainment has not been previously reported. In previous literature, high inherent alcohol tolerance in adolescence has been found to be associated with subsequent AUD and all-cause mortality in early adulthood, as well as self-harm and suicide [3, 4, 7, 18]. A number of factors likely impact an adolescents’ subjective experience of alcohol tolerance. Sex, weight and height are relevant, as are subjective perceptions of what “being intoxicated” means. Due to the participants’ young age, it is plausible that this self-report at least in part reflects some individual intrinsic characteristic (i.e., a trait) as 15/16-year-old adolescents have rarely had prolonged exposure to alcohol. There is a substantial amount of previous evidence pointing to lower responsiveness to alcohol’s effects among individuals with a family history of AUD [35]. It is plausible that this low responsiveness to alcohol with regard to subjectively experienced intoxication among the adolescents who report needing large amounts of alcohol in order to achieve intoxication, is indeed a trait feature and likely reflects a significant genetic predisposition for alcohol related problems. High inherent tolerance may be an early warning sign for subsequent development of heavy alcohol use or even AUD early on in life, which may in turn be a significant contributor to poorer academic achievements.

The associations between frequency of intoxication in adolescence and educational attainment were also statistically significant after controlling for confounders. Further, a dose-response relationship was found for frequency of intoxication, where more frequent instances of intoxication at age 15/16 years resulted in lower ORs for college/university education. This is in line with previous results from a national UK birth cohort where frequent binge drinking during adolescence has been found to predict negative educational outcomes, such as exclusion from school and leaving school without any qualifications [17].

Lifetime cannabis use at age 15/16 was not associated with educational attainment by age 33 after a range of confounders including frequency of self-reported intoxications were controlled for. Previously, in a British birth cohort, cannabis use at age 15 years has been found to be associated with adverse educational outcomes at age 16 years with a dose-response relationship [13]. Further, data from three Australasian birth cohorts showed adolescent cannabis use was associated with 1½ to two-fold increases in the odds of high-school non-completion, university non-enrolment and degree non-attainment [15]. It is to be noted that cannabis use in our cohort was relatively rare and infrequent with only 1.0% reporting lifetime use of five or more times. This may indicate that among this cohort, cannabis use overlaps with other risk factors, such as high frequency binge drinking, which are relevant in educational achievement later in life which may explain the discrepancies with results from previous birth cohort studies. It is also to be noted, that the cannabis use variable is not able to identify frequent e.g., weekly cannabis use, and heavier cannabis use is than classified in this study (at least 5 times by age 15/16 years) required to impair academic performance.

This study has several strengths. The NFBC 1986 is one of the largest birth cohort studies with high genetic and ethnic homogeneity. The study utilized several nationwide registers, providing data on diagnoses with low attrition. Attrition concerning those not participating the study at age 15/16 years is not a significant source of bias based on the additional analyses using inverse probability weighting. The wide range of prospectively collected information made it possible to address many potential confounders, including individual factors, such as early learning difficulties and psychiatric disorders, as well as family-related issues, such as maternal and paternal education and parental psychiatric disorders. However, inverse causality between the studied variables, such as family type and adolescent substance use, cannot be ruled out, which could suppress some of the effects seen in the multivariable models. The inclusion of four different alcohol related predictors can also be considered a strength of this study. It is to be noted, that all of the alcohol and cannabis use related variables were highly intercorrelated.

The information on substance use was collected using self-reports, which is subject to bias. Self-reports typically underestimate substance use [36] and may lead to underestimation of true associations. Information on lifetime alcohol and cannabis use was collected using self-reports at one time-point and we were not able to account for differential follow-up due to AFD or AFI. We were unable to differentiate between participants who remained abstinent from age 15/16 through age 33 from those who’s true AFD/AFI is older than age 16. The list of alcohol types used to determine AFD did not include ciders or long drinks, which may bias the results especially for female adolescents, who tend to prefer these beverages [37]. In addition, also inherent alcohol tolerance was assessed through self-report. Whether higher self-reported alcohol tolerance in fact can be interpreted as an individuals’ trait or rather as an acquired feature i.e., a result of alcohol exposure, could not be determined. It should also be noted that the data regarding alcohol and substance use was collected over 20 years ago and may not fully reflect the current trends in alcohol and substance use seen among today’s adolescents. Cannabis use was relatively rare, which could lead to power issues in the multivariable models. Despite these limitations, this study was able to robustly examine novel questions regarding early life alcohol and cannabis exposure and subsequent educational attainment taking into account a wide range of confounders.

## Conclusions

Our findings indicate that assessments of age of first alcohol intoxication, high self-reported alcohol tolerance and frequency of intoxication during adolescence should be included when implementing screening strategies aimed at identifying adolescents at risk for subsequent social problems e.g., poorer educational attainment. Alongside with systematic screening, implementation of adolescent alcohol use brief interventions should be implemented. As adolescent alcohol use seems to have life-long impact on educational attainment, also regulatory policies and multifaceted developmental interventions need be implemented to reduce the harm consequent on adolescent alcohol use.

### Electronic supplementary material

Below is the link to the electronic supplementary material.

Supplementary Material 1

## Data Availability

NFBC data are available from the University of Oulu, Infrastructure for Population Studies. Permission to use the data can be applied for research purposes via an electronic material request portal. In the use of data, we follow the EU general data protection regulation (679/2016) and the Finnish Data Protection Act. The use of personal data is based on a cohort participant’s written informed consent in their latest follow-up study, which may cause limitations to its use. Please, contact the NFBC project center for more information: https://www.oulu.fi/en/university/faculties-and-units/faculty-medicine/northern-finland-birth-cohorts-and-arctic-biobank/nfbc-aineistopyynto (data referral).

## Abbreviations

95% CI: 95% confidence interval
AFD: Age at first drink
AFI: Age at first intoxication
AUD: Alcohol use disorder
HED: Heavy episodic drinking
ICD-10: The International Classification of Disease, 10th revision
NFBC1986: The Northern Finland Birth Cohort Study 1986
OR: Odds ratio
SUD: Substance use disorder
