# Supplementary material for: Adolescent alcohol and cannabis use and early adulthood educational attainment in the 1986 Northern Finland birth cohort study
Source: BMC Public Health. 2024 Jan 22;24:255. doi: 10.1186/s12889-024-17693-w (PMC10804574; doi:10.1186/s12889-024-17693-w)
Supplement: Supplementary file 1 — Supplementary Material 1 [file 12889_2024_17693_MOESM1_ESM.docx]

**Supplement 1. Flowchart of the study.**

Information on

any cannabis use by age 15/16

n = 6586 (69.8%)

Information on

self-reported alcohol tolerance at age 15/16

n = 6615 (70.1%)

Information on

frequency of intoxication at age 15/16

n = 6462 (68.5%)

Information on

age at first intoxication

n = 6565 (69.6%)

Information on

age at first drink

n = 6631 (70.3%)

Excluded from analyses if deceased or immigrated

n = 391

Northern Finland Birth Cohort of 1986

All alive born children between 1/7/1985 – 30/6/1986

n = 9432

Register-based data on educational level at age 33 available

n = 7760 (82.3%)

Follow-up of participants at age 15/16

Participants with known address:

n = 9215 (97.2%)
